# High bone turnover in critically vitamin-D-deficient patients

**DOI:** 10.1186/cc9792

**Published:** 2011-03-11

**Authors:** K Amrein, H Sourij, A Holl, C Schnedl, TR Pieber, H Dobnig

**Affiliations:** 1 Medical University of Graz, Austria

## Introduction

Vitamin D deficiency, hypocalcemia and acute immobilization negatively affect bone metabolism and are present in the majority of critically ill patients. Although high bone turnover is highly prevalent in the ICU and might compromise long-term outcome, there are currently no data on fracture risk after critical illness.

## Methods

We assessed bone turnover comparing placebo (P) with a cholecalciferol loading dose (VITD) over a 1-week observation period in critically ill medical patients with vitamin D deficiency (25(OH)D ≤20 ng/ml). Markers of bone and mineral metabolism (β-CTx, 0.06 to 0.35 ng/ml, C-terminal telopeptide of type I collagen; OC, osteocalcin, 1.0 to 35.0 ng/ml) were analysed. Analyses were repeated at days 3 and 7 after 540,000 IU cholecalciferol or matched placebo were given enterally.

## Results

Twenty-five critically ill patients with an expected ICU stay of more than 48 hours were included (76% male, age 62 ± 16 years, 84% mechanically ventilated). Bone turnover was accelerated indicating bone loss and further deteriorated during the ICU stay. Calcium levels increased significantly in the vitamin D group only (Table 1), the mean serum 25(OH)D increase in the intervention group was 25 ng/ml.

**Table 1:** Biochemical markers of bone turnover (7-day observation)

	Day 0	Day 3	Day 7
**β-CTx**			
P	0.68	0.89	0.97*
VITD	0.57	0.76	0.81*
**OC**			
P	13.7	15.1	13.9
VITD	17.9	19.9	20.3
**Ca ion**			
P	1.02	1.04	1.09
VITD	1.07	1.13*	1.17*

*P < 0.05.

## Conclusions

Increased bone resorption is frequent in patients in the medical ICU. Intravenous bisphosphonates have been suggested to mitigate bone loss in patients at risk; however, correction of vitamin D deficiency might be a prerequisite for optimal efficacy in this vulnerable population.

